# Knock out CD44 in reprogrammed liver cancer cell C3A increases CSCs stemness and promotes differentiation

**DOI:** 10.18632/oncotarget.6090

**Published:** 2015-10-22

**Authors:** Shuo Han, Jinhai Guo, Yinan Liu, Zhi Zhang, Qihua He, Peng Li, Mingzhi Zhang, Haojie Sun, Ruizhi Li, Yang Li, Wotan Zeng, Jinwen Liu, Lejian Lian, Yi Gao, Li Shen

**Affiliations:** ^1^ Department of Cell Biology, Stem Cell Research Center, Department of Basic Medical Sciences, Peking University Health Science Center, Beijing, People's Republic of China; ^2^ Beijing DongFang YaMei Gene Science and Technology Research Institute, Beijing, People's Republic of China; ^3^ State Key Laboratory of Organ Failure Research, Co-Innovation Center for Organ Failure Research, Guangdong Provincial Research Center of Artificial Organ and Tissue Engineering, Second Department of Hepatobiliary Surgery, ZhuJiang Hospital, Southern Medical University, Guangzhou, People's Republic of China

**Keywords:** induced liver cancer stem cells, CD44, transcriptional regulation, CRISPR/Cas9, C3A

## Abstract

CD44 is a widely known cancer stem cells marker in various cancers and validated to function in tumor growth, survival and tumor metastasis. In this study, we first established C3A-derived liver cancer stem cells by OSKM method [OCT4, SOX2, KLF4, and c-MYC], termed C3A-induced cancer stem cells (C3A-iCSCs) which acquired self-renewal and stemness abilities. Then we found CD44 was positive in C3A-iCSCs and mainly located in cell nuclear. Chromatin immunoprecipitation-quantitative PCR (ChIP-qPCR) results showed nuclear CD44 combined promoter regions of c-*MYC* and *SOX2*. These results suggested that CD44 participated in C3A-iCSCs transcriptional regulation. To explore CD44 overall influence in liver cancer stem cells, CD44 was knocked out in C3A-iCSCs using CRISPR/Cas9 technology. Our results showed a dramatic increase in the expression of stem cell markers OCT4, SOX2 and NANOG in CD44^−^ C3A-iCSCs compared with that in CD44^+^ C3A-iCSCs. Tumor derived from CD44^−^ C3A-iCSCs also displayed well-differentiated tumor cells compared to CD44^+^ C3A-iCSCs, which suggested CD44^−^ C3A-iCSCs derived tumor cells exhibited lower malignant degree. Our data indicated nuclear CD44 in liver cancer stem cells is responsible for the poorly differentiated highly malignant tumor cells by maintenance of low stemness state.

## INTRODUCTION

Since Yamanaka developed reprogramming of somatic cells to induced pluripotent stem cells (iPS cells), it has been widely believed that iPS cells will become the convenient platform for disease modeling and therapies for different kinds of disease [1]. More attention has focused on cancer cells reprogramming to construct cancer stem cells (CSCs) models. CSCs are vital to cancer research. At present, the source and evolution of CSCs are still controversial. Cancer cells reprogramming could exhibit cancer progression, from early to late stages [2]. In the process of *in vitro* simulation of tumor development, markers of different stages and dynamic changes of signal pathways in tumor development will be clear and recognizable.

Liver cancer is the fifth most common cancer around the world [3]. Liver cancer development share similar features with liver development, including the development and maintenance of stem cells [4]. Liver CSCs indicate a subset of cells with self-renewal and possess stemness properties, these properties may contribute to metastatic, drug resistance and radiation resistance, in addition liver CSCs result in liver cancer heterogeneous phenotypes. CSCs are marker-positive, liver CSCs markers include CD13, CD24, CD44, CD90, CD133 and EpCAM, some of these markers are responsible for tumor highly invasive features and drug resistance [5, 6].

Among the liver CSCs markers, CD44 mainly assist other markers to isolate liver CSCs [5, 7]. A CD44 variant was reported to influence the redox status to protect CSCs from oxidative stress in liver cancer [8]. Actually, CD44 is widely known as a CSCs marker, not only in liver cancer but also in gastric cancer, breast cancer, acute myeloid leukemia [9–12]. Glycoprotein CD44 locates on the cell surface, which is involved in intercellular interactions, cell adhesion and migration. Alternative splicing of CD44 mRNA produces multiple isoforms with different functions. CD44 can be detected in the process of lymphocyte activation, recycling and homing, cancer development and metastasis.

In this study, we chose the human hepatocellular carcinoma cell line C3A derived from HepG2. The four Yamanaka factors OSKM were transfected into C3A cells. Then we successfully got C3A derived liver CSCs model that were subsequently termed C3A-induced cancer stem cells (C3A-iCSCs). C3A-iCSCs were identified CD44 positive and CD133 negative. CD133^−^CD44^+^ C3A-iCSCs displayed self-renew and stemness characters compared to CD133^+^CD44^−^ C3A cells. We found CD44 located mainly in nucleus of C3A-iCSCs and bound to promoter regions of tumor associated gene c-*MYC* and stem cell marker *SOX2*. To explore overall function of CD44 gene in liver cancer stem cells we knocked out CD44 in C3A-iCSCs using the CRISPR/Cas9 system. CD44^−^ C3A-iCSCs comparing with CD44^+^ C3A-iCSCs displayed higher level of stemness and increased tumor cellular differentiation after xenografts in mice. Our study validated CD44 have vital function in liver cancer stem cells through maintaining poorly differentiated tumor cells population. This finding suggests CD44 could be an important research object in the future in liver cancer therapy.

## RESULTS

### C3A-iCSCs were generated by OSKM transduction and possessed cancer stem-like properties

**Figure 1:**
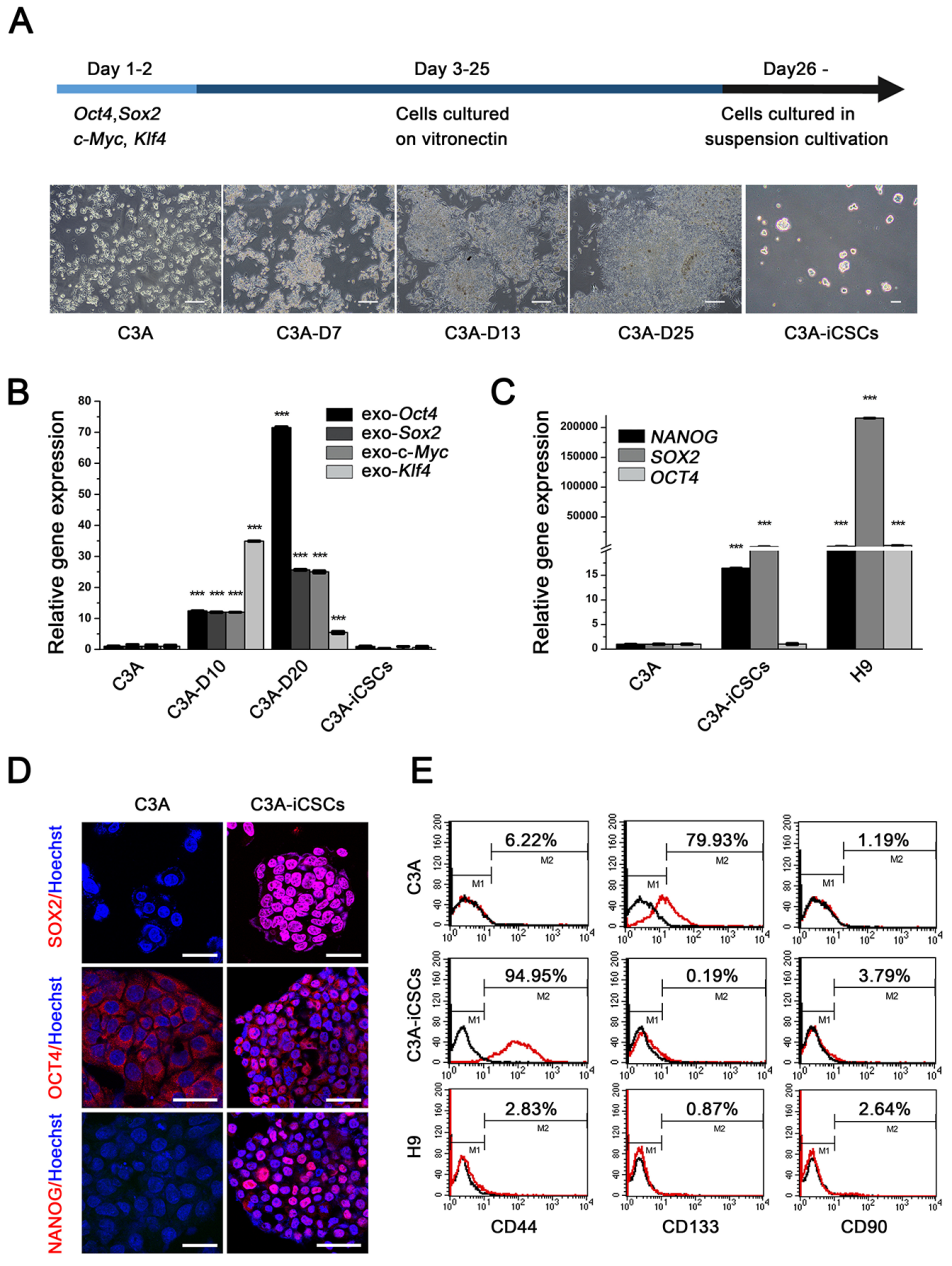
OSKM reprogramming of C3A cells into C3A-iCSCs **A.** Schedule of C3A cells reprogramming into C3A-iCSCs and changes in morphology during cell reprogramming. Morphology of C3A cells, C3A cells induced separately on day 7, day 13 and day 25 (C3A-D7, C3A-D13, C3A-D25), and C3A-iCSCs were recorded. Scale bar, 100 μm. **B.** Real-time PCR analysis of the exogenous *Oct4*, *Sox2*, *Klf4* and *c-Myc* expression in C3A cells, C3A-D10, C3A-D20 and C3A-iCSCs. Relative gene expression to C3A cells was calculated for C3A-D10, C3A-D20 and C3A-iCSCs and presented in the bar graphs with standard deviations. **C.** Real-time PCR analysis of the endogenous stem cell markers *OCT4*, *SOX2* and *NANOG* expression in C3A cells, C3A-iCSCs and H9 cells. Relative gene expression to C3A cells was calculated for C3A-iCSCs and H9 cells and presented in the bar graphs with standard deviations. **D.** Immunofluorescence staining of stem cell markers SOX2, OCT4 and NANOG in C3A cells and C3A-iCSCs. Red indicated positive staining. Nuclei were counterstained with Hoechst 33342 (blue). Scale bar, 40 μm. **E.** Flow cytometric analysis of liver CSC markers CD44, CD133 and CD90 in C3A cells and C3A-iCSCs. Number indicate the percentage of positive cells.

C3A cells were transduced with individual lentiviruses carrying *Sox2, Oct4, Klf4* and c-*Myc*. On the day 7, we observed a remarkable high nucleus-cytoplasm ration in individual cell, cells showed aggregate growth. On the day 13, colonies with clearly boundaries were composed of small, tight cells which were remarkably different from C3A cells. On day 26, to imitate the tumor cells metastasize, colonies were passaged in suspension culture condition, adherent colonies gathered into suspended cell spheres (Fig. 1A). Single cells sphere were picked up and expanded. These cell spheres were named as C3A-induced cancer stem cells (C3A-iCSCs).

Firstly, we assessed stemness state. After reprogramming, exogenous OSKM expression silenced in C3A-iCSCs (Fig. 1B), while expression of endogenous stem cell markers *SOX2* and *NANOG* increased, especially *SOX2*, which increased by 100-fold in C3A-iCSCs compared with C3A cells (Fig. 1C). Although *OCT4* expression level in C3A-iCSCs was similar to C3A cells, immunofluorescence analyses indicated that OCT4 located in the cytoplasm of C3A cells while OCT4 strongly expressed in the nucleus of C3A-iCSCs (Fig. 1D). OCT4 represents stemness level and expresses both in stem cells and CSCs. It functions to maintain stemness state [13]. Ectopic expression of OCT4 can be detected in cancer cells from tumor tissues [14]. To distinguish cancer stem cells and embryonic stem cells characters, H9 cells line was control group in the next series of experiments. Gene expression level of *OCT4*, *SOX2* and *NANOG* in C3A-iCSCs were lower compared to H9 cells (Fig. 1C), this data suggested C3A-iCSCs stemness state did not reach the level of H9. Next, we chose three liver CSCs markers CD44, CD133 and CD90 to examine liver CSCs characters in C3A-iCSCs, Flow cytometric analysis showed no expression of CD90 in both C3A-iCSCs and parental C3A cells. Expression of CD133 reached 79.93 ± 0.35% in parental C3A cells, which was in contrast to 0.19 ± 0.02% in C3A-iCSCs. CD44 expression was as much as 94.95 ± 0.23% in C3A-iCSCs and only 6.22 ± 0.46% in C3A cells, all three markers in H9 cells maintained silence (Fig. 1E). Therefore, we have primarily acquired liver cancer stem cells model using Yamanaka factors.

### C3A-iCSCs lose hepatocellular phenotypes and acquired pluripotent to differentiate to different kinds of cell types

**Figure 2:**
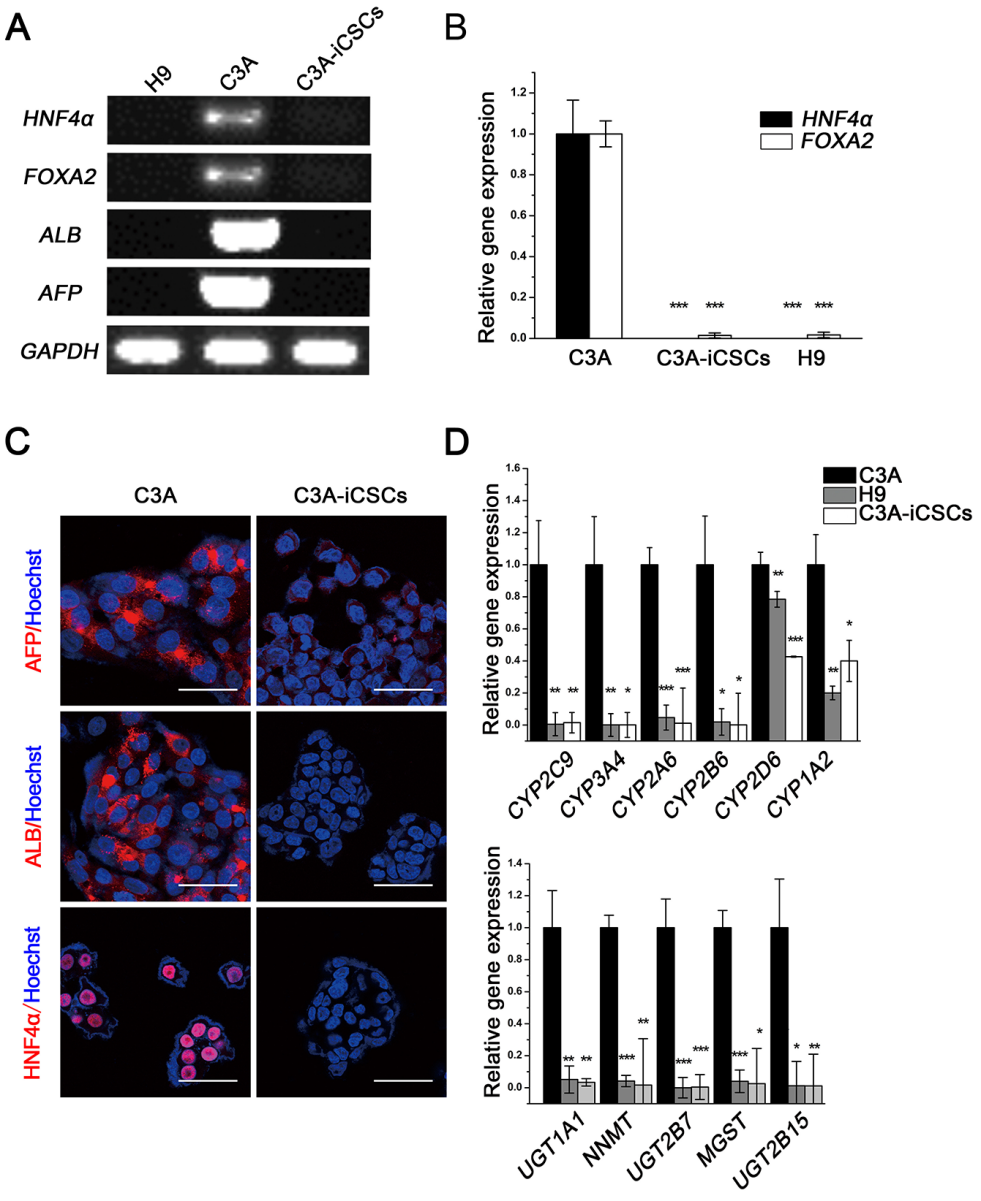
Hepatocyte phenotypes silenced in C3A-iCSCs **A.** RT-PCR analysis of liver-specific gene expression in C3A cells, C3A-iCSCs and H9 cells. **B.** Real-time PCR analysis of the liver transcription factors *HNF4α* and *FOXA2* expression in C3A cells, C3A-iCSCs and H9 cells. Relative gene expression to C3A cells was calculated for C3A-iCSCs and H9 cells and presented in the bar graphs with standard deviations. **C.** Immunofluorescence staining of ALB, AFP and HNF4α in C3A cells and C3A-iCSCs. Red indicates positive staining. Nuclei were counterstained with Hoechst 33342 (blue). Scale bar, 50 μm. **D.** Real-time PCR analysis of the drug metabolic enzyme gene expression in C3A cells, C3A-iCSCs and H9 cells. Relative gene expression to C3A cells was calculated for C3A-iCSCs and H9 cells and presented in the bar graphs with standard deviations. The upper graph showed results for phase I enzymes and the lower graph showed results for phase II enzymes.

It is reported that CSCs will lose their original cell-specific phenotype [15], we then examined whether C3A-iCSCs maintained the phenotypes of hepatoma carcinoma cells. We chose several markers of liver development at various stages (definitive endoderm marker *FOXA2*, hepatoblast marker *AFP*, further hepatic differentiation marker *ALB* and hepatic progenitor cell marker *HNF4α*). RT-PCR showed that they expressed in C3A cells but silenced in C3A-iCSCs (Fig. 2A). Real-time PCR and immunofluorescence analyses confirmed the same results (Fig. 2B and 2C). Drug metabolism-associated genes were decreased in C3A-iCSCs compared with C3A cells (Fig. 2D). These data demonstrated that C3A-iCSCs had lost the phenotypes of hepatoma carcinoma cells.

**Figure 3:**
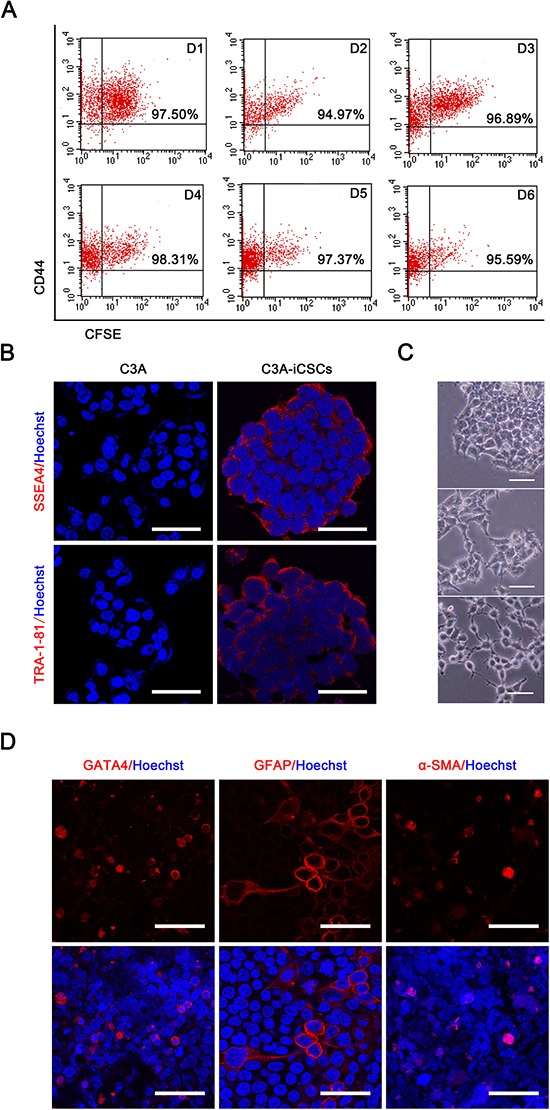
C3A-iCSCs acquired self-renewal and pluripotency **A.** Flow cytometric analysis of CD44 and Carboxyfluorescein succinimidyl ester on C3A-iCSCs from day 1 to 6 (D1-D6). Number indicated the percentage of CD44-positive cells. **B.** Immunofluorescence staining of pluripotent markers SSEA4 and TRA-1–81 in C3A cells and C3A-iCSCs. Red indicated positive staining. Nuclei were counterstained with Hoechst 33342 (blue). Scale bar, 40 μm **C.** Representative phase-contrast images of the different morphology of differentiated C3A-iCSCs. C3A-iCSCs were cultured on gelatin-coated plates for 7 days. Scale bar, 50 μm. **D.** Immunofluorescence images of differentiated C3A-iCSCs stained for lineage-specific markers GATA4, GFAP and BRY. Red indicated positive staining. Nuclei were counterstained with Hoechst 33342 (blue). Scale bar, 40 μm.

Cancer stem cells possess the ability of self-renewal and give rise to all cell types in one tumor sample [16]. Carboxyfluorescein succinimidyl ester and CD44 antibody double labeling displayed stable CD44 expression in C3A-iCSCs with continuous passage, this showed that C3A-iCSCs had the ability to self-renewal (Fig. 3A). In C3A-iCSCs, pluripotent stem cell-specific markers SSEA-4 and TRA-1–81 were positive just like H9 cells which proved C3A-iCSCs had potential to differentiate (Fig. 3B). Then C3A-iCSCs were cultured in the differentiation condition for 7 days, a variety of cellular morphology appeared from C3A-iCSCs (Fig. 3C), these differentiated C3A-iCSCs were examined with different tissue-specific markers. Immunofluorescence analyses showed C3A-iCSCs had differentiated and expressed the endoderm marker GATA4, ectoderm marker GFAP, and mesoderm marker BRY (Fig. 3D). These data indicated that C3A-iCSCs possessed pluripotency.

### Nuclear CD44 combined promoter regions of tumor associate-gene c-*MYC* and stem cell gene *SOX2* in C3A-iCSCs

**Figure 4:**
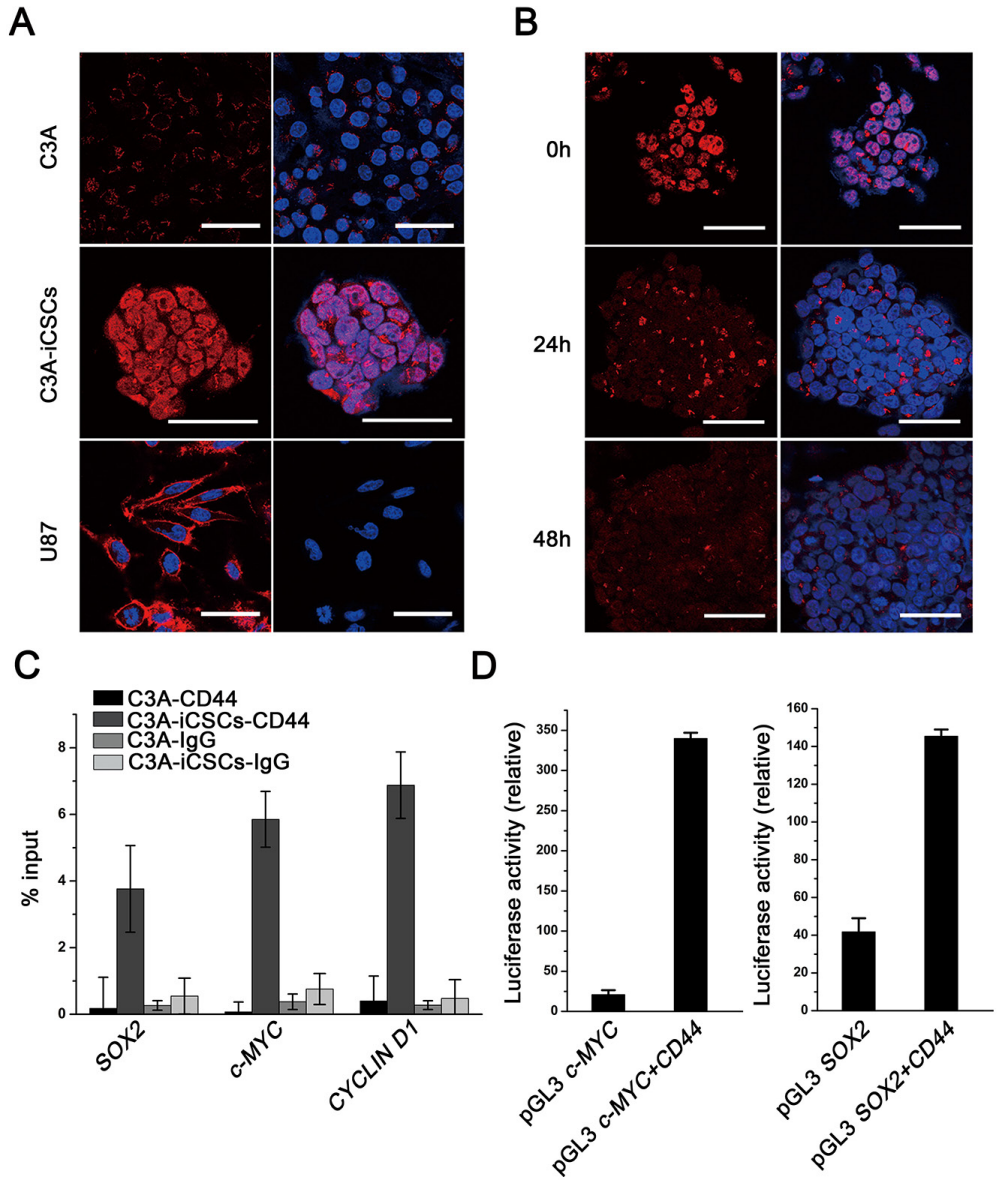
Nuclear CD44 participated in transcriptional regulation of C3A-iCSCs **A.** Immunofluorescence staining of CD44 in C3A cells, C3A-iCSCs and U87 cells. U87 cells were used as a positive control and C3A cells as a negative control. Red indicated positive staining. Nuclei were counterstained with Hoechst 33342 (blue). Scale bar, 40 μm. **B.** Immunofluorescence staining of CD44 expression in C3A-iCSCs and C3A-iCSCs cultured in DMEM containing 10% fetal bovine serum medium for 24 and 48 h. Red indicated positive staining. Nuclei were counterstained with Hoechst 33342 (blue). Scale bar, 40 μm. **C.** ChIP-qPCR using an anti-CD44 antibody and anti-IgG control in C3A cells and C3A-iCSCs. The *CYCLIN D1* promoter was used as a positive control which have been reported that CD44 protein could bound to [17]. *SOX2, c-MYC* and *CYCLIN D1* represented respectively their promoters. Samples were analyzed by qPCR. Error bars showed the standard deviation of three independent ChIP-qPCR assays. **D.** Luciferase activity assay about CD44 transcriptional regulation ability. Luciferase reporter plasmids pGL3 *c-MYC* promoter and pGL3 *SOX2* promoter were co-transfected with the pCMV3-*CD44* plasmid and Renilla control vector in 293T cells. Luciferase activities were measured after 48 h and presented as relative to the activity of Renilla luciferase. pGL3 *SOX2* promoter and pGL3 *c-MYC* promoter groups were control.

Protein functions are closely connected with cellular localization. CD44 is a cell-surface glycoprotein. Through binding with hyaluronic acid and other ligands CD44 functions in cell-cell interaction, cell adhesion and migration. While recently it has been reported that CD44 can translocate to the nucleus, full-length CD44 enters the nucleus by binding with nuclear import proteins and plays a functional role in cell proliferation and survival [17]. We first detected CD44 location in C3A-iCSCs before explore CD44 function in C3A-iCSCs. Immunofluorescence analyses showed CD44 mainly located in nucleus and there was a little in cytoplasm (Fig. 4A). To detect the sensitivity of CD44 marker, C3A-iCSCs were cultured under cell differentiated condition, with the duration of differentiation time CD44 expression immediately and gradually declined (Fig. 4B).

Lee and colleagues found that the standard CD44 expressed in the nucleus could reprogram colon cancer cells line to CSCs under suspension culture, CD44 could integrate with STAT3 and gp130 after reprogramming and function as a transcription factor complex [18]. To verify whether nuclear CD44 participated in the liver cancer stem cells transcriptional regulation, chromatin immunoprecipitation-quantitative PCR (ChIP-qPCR) assay was performed. Cancer-related gene *c-MYC* promoter was validated to combine with CD44 in colon cancer stem cells [19]; *SOX2* was chosen for its indispensable position in various CSCs [20, 21]. ChIP-qPCR results showed that nuclear CD44 in C3A-iCSCs bound to promoter regions of *SOX2* and c-*MYC* in C3A-iCSCs (Fig. 4C). Luciferase assay reconfirmed these findings, we found that CD44 indeed bound to the promoter regions of *SOX2* and *c-MYC* in 293T cells (Fig. 4D). These data suggest nuclear CD44 participate in cancer-related and stemness-related transcriptional regulation in liver CSCs.

### CD44 was knocked out in C3A-iCSCs using the CRISPR/Cas9 system

**Figure 5:**
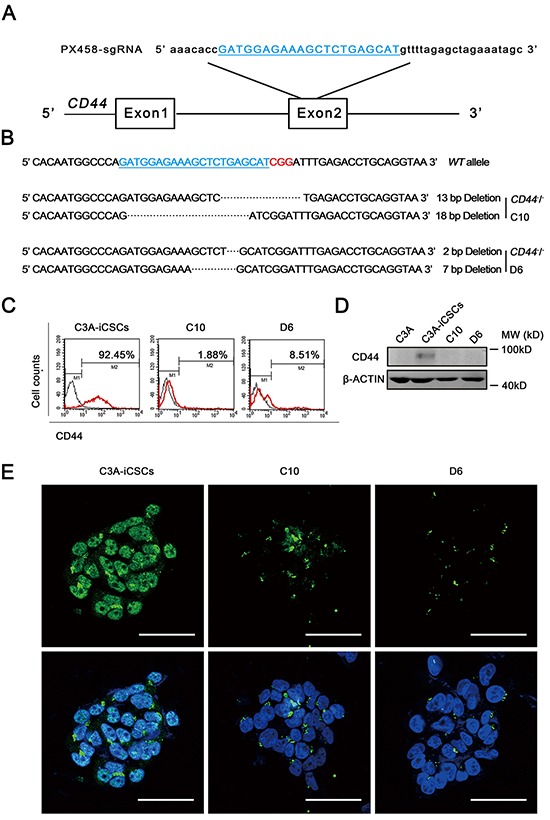
Generation of CD44 knockout C3A-iCSCs using CRISPR/Cas9 system **A.** The sgRNA (blue) was designed to aim at 20 pairs of bases in exon 2 of *CD44*. **B.** Sequences of the wild-type (WT) CD44 locus and DSBs induced by Cas9 of CD44 locus in two established cell lines (CD44-KO C3A-iCSCs D6) D6 and (CD44-KO C3A-iCSCs C10). C10 had one allele with a 13 bp deletion and the other with an 18 bp deletion. D6 had one allele with a 2 bp deletion and the other with a 7 bp deletion. The PAM sequence was indicated by red color. **C.** Flow cytometric analysis of CD44 expression in C3A-iCSCs, D6 and C10 cells. Number indicated the percentage of CD44-positive cells. **D.** Western blotting of CD44 in C3A-iCSCs, C3A, D6 and C10 cells, MW: molecular weight of markers. **E.** Immunofluorescence staining of CD44 in C3A-iCSCs, D6 and C10 cells. Green indicated positive staining. Nuclei were counterstained with Hoechst 33342 (blue). Scale bar, 50 μm.

As CD44 has been proved to participate in the transcriptional regulatory network in C3A-iCSCs, we knocked out CD44 in C3A-iCSCs to further explore the overall influence of CD44 in liver CSCs. We used the CRISPR/Cas9 system that had been reported to efficiently disrupt gene in various organisms [22]. As a result, we obtained two CD44 knockout C3A-iCSCs clones named CD44^−^ C3A-iCSCs D6 clone (D6) and CD44^−^ C3A-iCSCs C10 clone (C10). DNA sequencing of C10 cells revealed deletion of 18 and 13 bases in both alleles, and D6 cells had deletion of 2 and 7 bases in both alleles (Fig. 5A and 5B). Western blotting, flow cytometry and immunofluorescence showed that CD44 was completely knocked out in D6 and C10 cells by an absence of CD44 expression (Fig. 5C–5E).

### CD44^−^ C3A-iCSCs acquired higher stemness state and promoted differentiation *in vivo*

**Figure 6:**
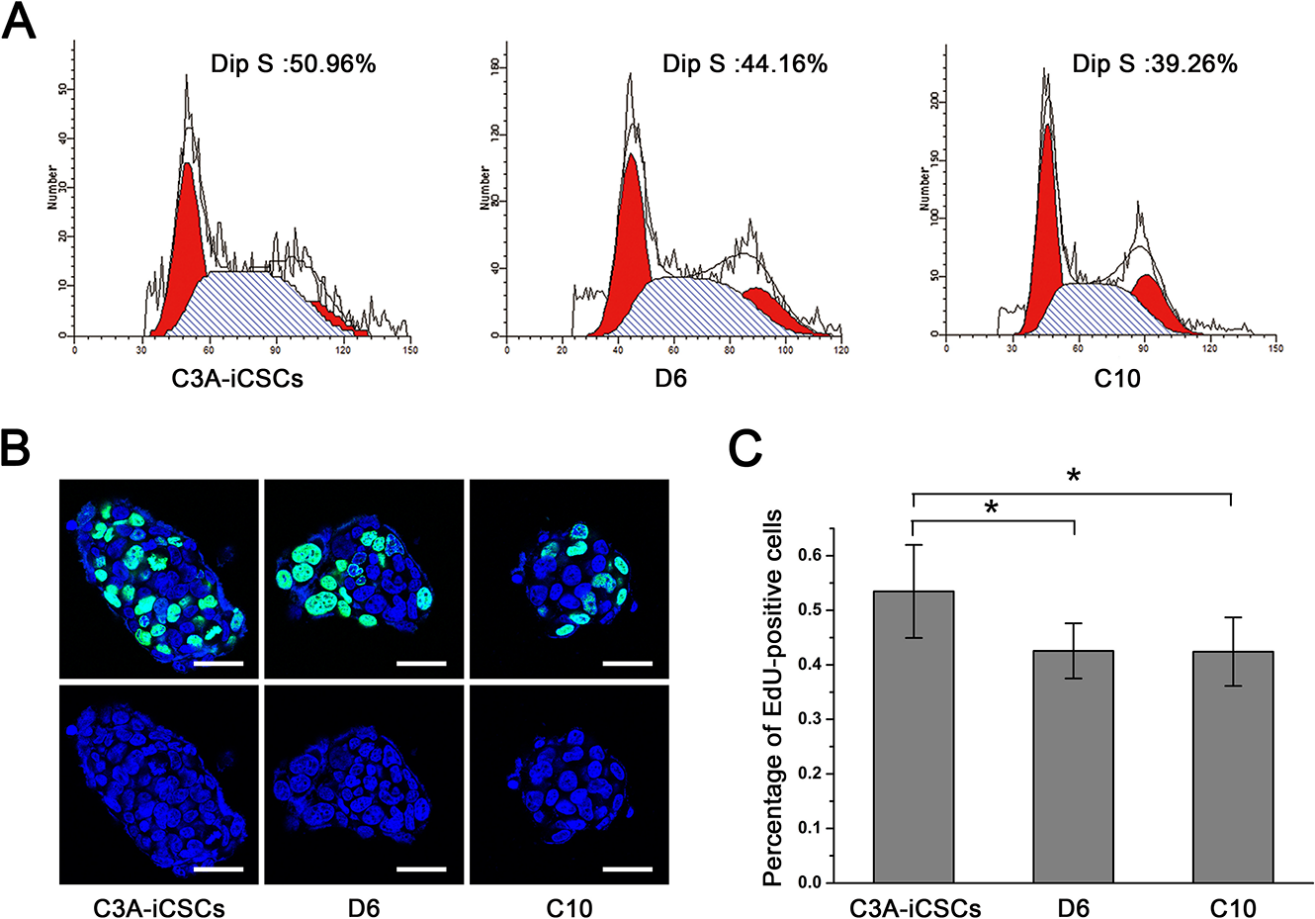
CD44^−^ C3A-iCSCs got lower proliferation **A.** Flow cytometric analysis of the cell cycle of C3A-iCSCs, D6 and C10 cells. Numbers indicated the percentage of cells in S phase. **B.** Immunofluorescence staining of EdU positive cells of C3A-iCSCs, D6 and C10 cells. Green indicated positive staining. Nuclei were counterstained with Hoechst 33342 (blue). Scale bar, 40 μm. **C.** Histogram of percentage of EdU positive cells in C3A-iCSCs, D6 and C10 cells with standard deviations. *P* < 0.05, *n* = 6.

**Figure 7:**
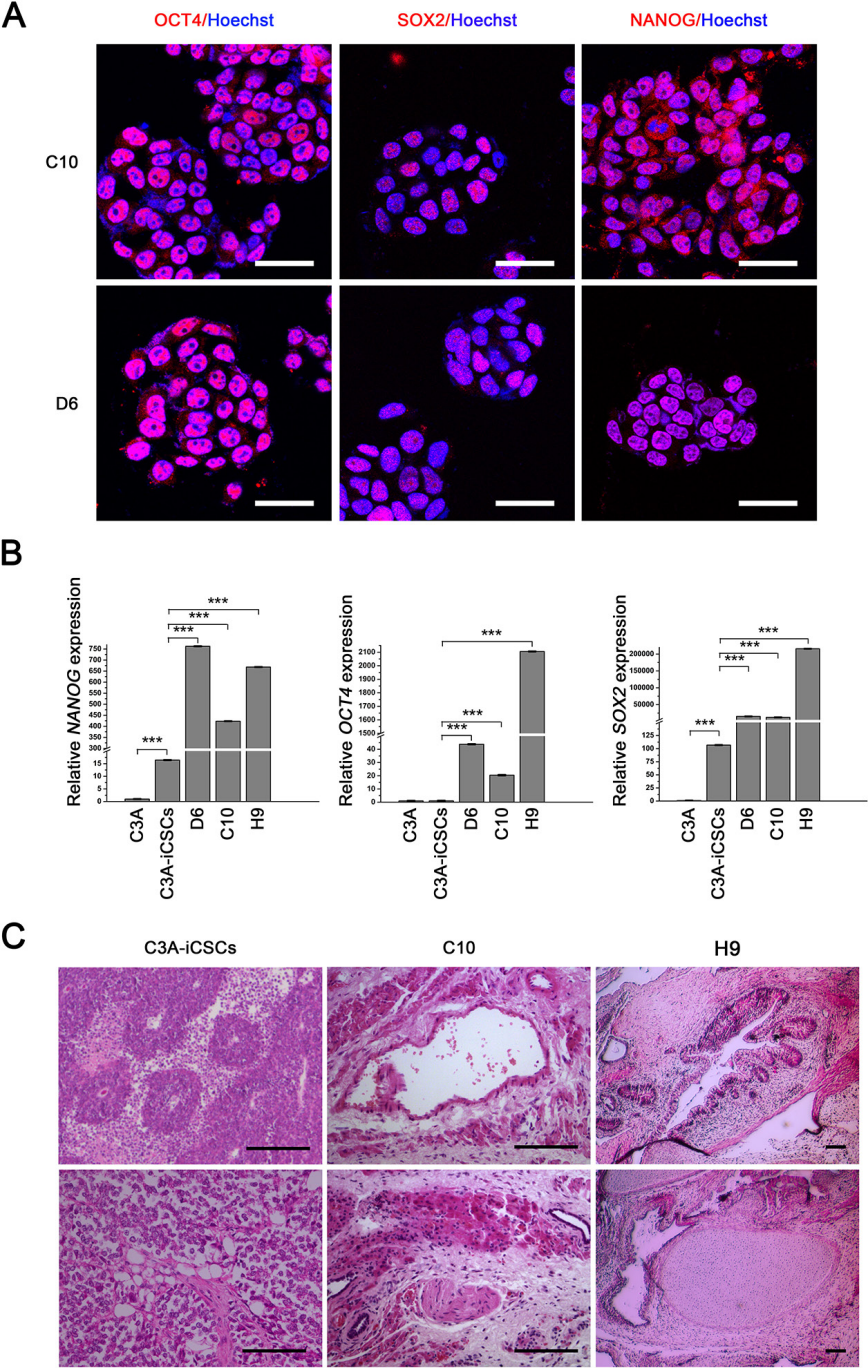
CD44^−^ C3A-iCSCs acquired a greater capacity of pluripotency **A.** Immunofluorescence staining of OCT4, SOX2 and NANOG in D6 and C10 cells. Red indicated positive staining. Nuclei were counterstained with Hoechst 33342 (blue). Scale bar, 40 μm. **B.** Real-time PCR analysis of *OCT4*, *SOX2* and *NANOG* expression in C3A-iCSCs, C3A, D6, C10 and H9 cells. Relative gene expression to C3A cells was calculated for C3A-iCSCs, C3A, D6, C10 and H9 cells and presented in the bar graphs with standard deviations. **C.** Hematoxylin and eosin (H&E) stained xenograft tumor sections derived from C3A-iCSCs, C10 cells and H&E stained xenograft teratoma sections derived from H9 cells. C3A-iCSCs derived tumor sections displayed muscle-like tissue, adipose-like cells and neural tubes-like structures. C10 cells derived tumor sections displayed gastrointestinal-like epithelium, neural tubes-like structures, muscle-like tissue. H9 cells derived teratoma sections displayed three-germ morphology including gastrointestinal epithelium, neural tubes-like structures, muscle and chondrocyte. Scale bar, 100 μm.

Next, we characterized the stemness of CD44^−^ C3A-iCSCs. Firstly, Analysis of the cell cycle by flow cytometry and nuclear EdU cell proliferation assay showed that D6 and C10 cells had a lower proliferative state than C3A-iCSCs (Fig. 6A–6C). So we speculated CD44 had assisted liver CSCs growth. Immunofluorescence images of OCT4, SOX2 and NANOG in D6 and C10 cells indicated CD44^−^ C3A-iCSCs maintained stemnesss state (Fig. 7A). However, real-time PCR displayed there were a dramatic increases of *SOX2* and *NANOG* expression in D6 and C10 cells, which increased over 100-fold; *OCT4* also increased 40-fold in D6 cells and 20-fold in C10 cells compared with C3A-iCSCs (Fig. 7B). This finding suggested CD44 may play as negative factor for regulating stemness in liver CSCs. It inspired us if C3A-iCSCs acquired great pluripotent ability. So CD44^−^ C3A-iCSCs and C3A-iCSCs were injected in nude mice respectively, 4–6 weeks later tumors were taken out. Hematoxylin and eosin (H&E) stained tumor tissue sections displayed that CD44^−^ C3A-iCSCs compared to CD44^+^ C3A-iCSCs exhibited well-differentiated tumor cells and contained more kinds of cell types, and CD44^−^ C3A-iCSCs derived tumor cells morphology resembled H9 cells derived teratoma with three-germ morphology (Fig. 7C). These data indicated that knock out CD44 in induced liver CSCs increases CSCs stemness and promotes differentiation to normal cells-like morphology.

## DISCUSSION

A small subset of cancer cells called cancer stem cells (CSCs) possess self-renewal ability and could give rise to new tumor. CSCs are responsible for tumorigenicity, metastasis, drug resistance and resistance to radiation therapy. Liver cancer is the second most frequent cause of cancer death in men worldwide [23]. Clinical research proved liver cancer histologically heterogeneous and containing CSCs [24–26]. While compared to other cancers like colon cancer, liver CSCs need to further explore especially by building models of cancer stem cells to further study the development and progression of cancer. In this study, we have applied a new protocol to establish human liver CSCs model using Yamanaka factors which was reported useful in breast cancer cells line MCF-7 [27]. In liver CSCs model, we not only found CD44 function importantly, this protocol can provide a platform to study liver cancer stem cells in therapeutic tumor treatment and evaluation of treatment efficacy.

Liver CSC markers include EpCAM, CD133, CD90, CD44, CD24, CD13, and OV6, some of these markers are considered functional with highly invasive, metastasis, chemotherapy resistant of liver cancer [6, 7, 28–32]. During the progress of induced liver CSC formation, we first discovered CD44 expressed in the liver CSCs while classic liver CSCs marker CD133 was silence. It reminds us whether CD44 represents a high level of stemness. Compared to CD133, CD44 is identified as a classic CSC marker and widely presents in various cancers including breast, stomach, glioma, colon, head and neck cancers [33–35], while CD133 mainly express in glioma, neural tumors and liver cancer. This difference may be attributed to that CD44 is a receptor for HA and other ECM components. by binding to HA and other ligands CD44 activates stemness-related pathways including Nanog-Stat3, Oct4-Sox2-Nanog. Therefore, CD44 positive liver CSCs may represent high stemness level population. In our study, we also discovered that CD44 mainly expressed in the nucleus of liver CSCs. CD44 had been reported to express strongly in the nucleus and cell membrane in gastric cancer [19]. CD44 binds with two import receptors of importin β superfamily, by transportin-dependent pathway and translocated to nuclear [16]. Following the CD44 nuclear expression, we found that CD44 directly involved in the transcriptional regulation of liver CSCs, although nuclear CD44 could bind to various promoters [18], overall function of CD44 in liver CSCs is not much clear.

We applied CRISPR/Cas9 system to knock out CD44. CRISPR/Cas9 is a convenient and effective gene editing technique. This technology makes genome engineering practical in short term and quickly obtain a stable genotype cell line. In this study, CD44^−^ liver CSCs displayed high level of stemnes state and got well-differentiated tumor. This suggests that CD44 positively correlate with malignant degree of liver cancer and negatively correlate with stemness state of liver CSCs. Coincidentally, cancer stem cells marker CD44 has been reported to represent a low level of stemness in induced pluripotent stem (iPS) cells. O'Malley and colleagues demonstrated that, in the course of reprogramming from mouse embryonic fibroblasts to iPS cells, fully reprogrammed iPS cells can be marked and screened by silencing of CD44 expression together with ICAM1 and NANOG increase expression [36]. In human iPS cells, CD44 was also demonstrated to be a negative marker in reprogramming [37]. Furthermore, differentiation degree of tumor cells determines tumor grade. CD44^−^ liver CSCs got well-differentiated tumor tissue, possibly because stemness state of CD44^−^ liver CSCs was more close to H9 cells. After knocking out CD44, liver CSCs exhibited lower tumor characters and higher stemness level, CD44^−^ liver CSCs then acquire phenotype similar to normal liver progenitor cells. Therefore, it suggests that nuclear CD44 could be used as potential drug screening objective on liver CSCs.

In summary, we applied a new protocol to establish liver CSCs model by using Yamanaka factors. We found CD44 mainly located in nuclear region and was responsible for the poorly differentiated highly malignant tumor cells by maintenance of low stenmness state. Therefore knock out CD44 in reprogrammed liver cancer cells C3A increases CSCs stemness and promotes differentiation

## MATERIALS AND METHODS

### Cell lines and culture

All cells were cultured at 37°C with 5% CO2. C3A cells were derived from the human hepatoma cell line HepG2. The culture medium for C3A cells was Eagle's minimum essential medium (Gibco, New York) containing 10% fetal bovine serum (HyClone, Logan), 0.1 mM non-essential amino acids (Gibco, New York), and 0.1 mM 2-mercaptoethanol (Gibco, New York). Culture medium for H9 cells was Dulbecco's modified Eagle's medium (DMEM)/Ham's F-12 medium (Gibco, New York) containing 20% knockout serum replacement (Gibco, New York), 1 mM L-glutamine, 0.1 mM nonessential amino acids, 0.1 mM 2-mercaptoethanol, and 10 ng/ml recombinant human basic fibroblast growth factor (bFGF; Life Technologies, New York). Suspension culture was H9 medium without bFGF. H9 cells were maintained on Matrigel (BD Biosciences, Franklin Lakes) and passaged with 0.5 mM EDTA. C3A-iCSCs were passaged with Accutase (Life Technologies, New York), C3A cells were detached with 0.5% trypsin/0.25% EDTA. In all experiments, cells were in P5-P20.

### Cell reprogramming

C3A cells were transduced with lentivirus vectors. Tet-O-FUW-*Pou5f1*, -*Sox2*, -*Klf4*, and -c-*Myc* lentivirus vectors (Addgene, Cambridge, USA) were designed with a tetracycline responsive element. The plasmids were co-transfected with pCMV-Gag-Pol and pCMV-VSVG into 293T cells using VigoFect transfection reagent (Vigorous Biotechnology, China). After 48 h, virus-containing supernatants were collected and filtered through a 0.45 μm filter. The viruses were then concentrated by ultracentrifugation and transduced into C3A cells in medium containing 10 ng/ml polybrene. C3A cells were incubated in virus/polybrene-containing supernatants for 8 h. The cells were then passaged onto vitronectin (Peprotech, Rocky Hill)-coated plates in C3A-iCSCs culture medium with 20 ng/ml doxycycline (SIGMA-ALDRICH, St. Louis) induction. Cells were maintained in culture for 25 days. On day 26, culture was changed to suspension culture. Then single cell sphere was picked up and passaged.

### *In vitro* differentiation of C3A-iCSCs

C3A-iCSCs were passaged onto gelatin-coated dishes and incubated in DMEM containing 10% fetal bovine for another 7 days. The cells were then stained for GATA4 (Cell Signaling, Danvers), GFAP (Cell Signaling, Danvers), and BRY (Cell Signaling, Danvers) by immunofluorescence.

### RNA isolation, RT-PCR, and semi-quantitative real-time PCR

Total RNA was extracted using Trizol (Life Technologies, New York). cDNA synthesis was performed with a M-MLV Reverse Transcriptase kit (Promega, Madison) in accordance with the manufacturer's instructions. RT-PCR was performed with ExTaq (Takara Bio). Real-time PCR was performed with GoTaq^®^ qPCR Master Mix (Promega, Madison). Primer sequences are listed in Table 1. Signals were detected with Mx3000P and Mx3005P QPCR Systems (Agilent Technologies, Santa Clara, USA).

**Table 1:** primers used for real-time PCR, RT-PCR, Chip-qPCR and luciferase assay

Gene symbol	Forward / Reverse primers	Primer sequences 5′-3′
endo *OCT4*	F	GGGAGATTGATAACTGGTGTGTT
	R	GTGTATATCCCAGGGTGATCCTC
endo *SOX2*	F	GGGAAATGGGAGGGGTGAAAAGAGG
	R	TTGCGTGAGTGTGGATGGGATTGGTG
endo *NANOG*	F	CTAAGAGGTGGCAGAAAAACA
	R	CTGGTGGTAGGAAGAGTAAAGG
exo *Oct4*	F	ATGGGGAAAGAAGCTCAGTG
	R	GGCATTAAAGCAGCGTATCC
exo *Sox2*	F	GCCCCTGTCGCACATGT
	R	GGCATTAAAGCAGCGTATCC
exo *Klf4*	F	TGCCTTACACATGAAGAGGCAC
	R	GGCATTAAAGCAGCGTATCC
exo *c-Myc*	F	ACAGCTTCGAAACTCTGGTGC
	R	GGCATTAAAGCAGCGTATCC
*HNF4α*	F	GAGGAACCAGTGCCGCTACT
	R	TCTGGACGGCTTCCTTCTTC
*FOXA2*	F	GCCTGAAGCCGTCGTCTT-4
	R	CCGCAGATACCTCCTACTACCA
*AFP*	F	TTTTGGGACCCGAACTTTCC
	R	CTCCTGGTATCCTTTAGCAACTCT
*ALB*	F	GGTGTTGATTGCCTTTGCTC
	R	CCCTTCATCCCGAAGTTCAT
*GAPDH*	F	GGAAGGTGAAGGTCGGAGTCA
	R	GTCATTGATGGCAACAATATCCACT
*UGT1A1*	F	GTTGATCCCAGTGGATGGCAG
	R	TGATGTACAACGAGGCGTCAG
*UGT2B7*	F	GCCAACGTAATTGCATCAGCC
	R	TTCCCATCAAATCTCCACAGAACCT
*UGT2B15*	F	CTGCCTAAGGAAATGGAAGAG
	R	CATGTTACTGATCATCGACCC
*MGST*	F	TGTATTCCTTGAGTGGTCCCG
	R	CTCCGACAAATAGTCTGAAGTGC
*NNMT*	F	TAGAGGCTGCTGTGAAAGAGG
	R	TGGAAGAATAACTTTGCGAGAT
*CYP2D6*	F	GTGTCCAACAGGAGATCGACG
	R	CACCTCATGAATCACGGCAGT
*CYP2C9*	F	GCCACATGCCCTACACAGATG
	R	GCCACATGCCCTACACAGATG
*CYP1A2*	F	CTTCGTAAACCAGTGGCAGG
	R	AGGGCTTGTTAATGGCAGTG
*CYP3A4*	F	AGCCTGGTGCTCCTCTATCT
	R	CCCTTATGGTAGGACAAAAT
*CYP2B6*	F	CCGGGGATATGGTGTGATCTT
	R	CCGAAGTCCCTCATAGTGGTC
*CYP2A6*	F	GAGTTCCTGTCACTGTTGCG
	R	GTCCTGGCAGGTGTTTCATC
Chip *SOX2*	F	TGTGGATGAGCGGGAGAACAAT
	R	GTGCAGGGTACTTAAATGAGGAT
Chip c-*MYC*	F	ACACATCTCAGGGCTAAACAG
	R	GCACAGCTATCTGGATTGGAT
Chip *CYCLIN D1*	F	GAAGATGCAGTCGCTGAGATTC
	R	GCGACACCCCATATCCAAGC
*SOX2* promoter (luciferase assay)	F	gg ggtacc GGGGGGAGTGCTGTGGATGAG
	R	ccc aagctt GCCTGGGGCTCAAACTTCTCT
*C-MYC* promoter(luciferase assay)	F	gg ggtacc CCCCTCCCATATTCTCCCGTC
	R	ccc aagctt CGTCTAAGCAGCTGCAAGGAG
*CYCLIN D1* promoter(luciferase assay)	F	gg ggtacc ATCCCTTTAACTTTTAGGGTTA
	R	ccc aagctt CGATCTTCCGCATGGACGGCAG

### Immunofluorescence

C3A-iCSCs, D6 and C10 cells were cultured on matrigel for 3 hours before formalin fixation. Permeabilization was performed with 10% Triton^®^ X-100 and blocking with 10% bovine serum albumin (SIGMA-ALDRICH, St. Louis). Primary antibodies against the following molecules were used: NANOG (Abcam, England), OCT4 (Santa Cruz Biotechnology, Dallas), SOX2, SSEA4 (Chemicon, Billerica), TRA-1–81 (Santa Cruz Biotechnology, Dallas), AFP (Abcam, England), ALB (Abcam, England), Hnf4α (Cell Signaling, Danvers), and CD44 (Proteintech, Rocky Hill). Secondary antibodies were Alexa Fluor^®^488/594goat anti-rabbit/mouse IgGs (Origene, Rockville). Counter staining was performed with Hoechst 33342. Images were captured under a TCSSP5 Confocal Microscope (Leica, Buffalo Grove, USA). Merged images were obtained according to the recommended procedure using the Leica software.

### Flow cytometry

Flow cytometry was performed according to protocols from Affymetrix. PE-Cy7^®^-conjugated CD44 (Life Technologies, New York), CD133/1(AC133)-PE (Miltenyi Blotec, Germany), and fluorescein-isothiocyanate-anti human CD90 (BD Biosciences, Franklin Lakes) antibodies as well as Carboxyfluorescein succinimidyl ester (Dojindo, Japan) were used according to the manufacturer's instructions. Signals were detected with a FACSCalibur flow cytometer (BD Biosciences, Franklin Lakes).

### Tumor and teratoma formation and histological analysis

Investigation has been conducted in accordance with the ethical standards and according to the Declaration of Helsinki and according to national and international guidelines and has been approved by the authors' institutional review board.

C3A-iCSCs, D6 and C10 cells, H9 cells were suspended at 1 × 10^7^ cells/ml in phosphate-buffered saline (PBS). A total of 100μl of the cell suspension (1 × 10^6^ cells) was subcutaneously injected into the inside of the lower limbs of nude mice. After 4–6 weeks, tumors were removed from the mice. The samples were weighed, fixed in PBS containing 4% formaldehyde, and embedded in paraffin. Sections were stained with hematoxylin and eosin (Origene, Rockville).

### Western blot analysis

The primary antibody used was an anti-CD44 polyclonal antibody (Proteintech, Chicago). Images were captured by the Odyssey Fluorescent Western Scanning System (LI-COR, Lincoln, USA). Data were analyzed with the Odyssey Imaging System.

### ChIP-qPCR

ChIP was performed following the Cross-linking chromatin immunoprecipitation protocol (http://www.abcam.cn) and as previously described [38]. The anti-CD44 polyclonal antibody was used in this experiment. Real-time PCR primers are listed in Table 1.

### Dual luciferase assay

293T cells were transfected using VigoFect transfection reagent. After 48 h, cells were lysed, and the firefly and Renilla luciferase activities were measured using Dual-Luciferase Reporter Assay System (Promega, Madison) according to the pGL3 Luciferase Reporter Vector Technical Manual (http://www.promega.com). *SOX2*, c-*MYC*, and *CYCLIN D1* promoter regions were amplified with specific primers and the fragments were ligated into pGL3-basic vectors (Promega, Madison). Primer sequences are listed in Table 1. The results are presented as the ratio of Renilla to firefly luciferase activities.

### *CD44* knockout by the CRISPR/Cas9 system

sgRNA were designed using the ZiFit Web application (http://zifit.partners.org/) to aim at exon 2 of *CD44*. The gene-specific sgRNA sequence was 5′ GATGGAGAAACTCTGAGCAT 3′. sgRNA was cloned into the px458 vector (Addgene, Cambridge, USA). Plasmid construction and isolation of clonal cell lines were conducted as described previously [39].

### EdU cell proliferation assay

EdU cell proliferation assay were performed using EdU HTS Kit 488 (SIGMA-ALDRICH, St. Louis). C3A-iCSCs, D6 and C10 cells were cultured on matrigel for 3 hours before formalin fixation.
